# Trends of sexual behaviors and related factors in high-risk MSM from 2010 to 2023: a repeated cross-sectional study in Zhejiang, China

**DOI:** 10.1186/s12889-025-25517-8

**Published:** 2025-12-18

**Authors:** Wenwen Liu, Xiaoxiao Chen, Shanling Wang, Yating Wang, Haijiang Lin, Ye Yao

**Affiliations:** 1 Shanghai Institute of Infectious Disease and Biosecurity, Fudan University, Shanghai, China; 2 Department of Epidemiology, School of Public Health, Fudan University, Shanghai, China; 3 Taizhou Blood Center, Taizhou, Zhejiang China; 4 Taizhou City Center for Disease Control and Prevention, Taizhou, Zhejiang China; 5 Department of Biostatistics, School of Public Health, Fudan University, Shanghai, China; 6 Key Laboratory of Public Health Safety of Ministry of Education, Fudan University, Shanghai, China

**Keywords:** MSM, High-risk MSM, Bisexual MSM, Active MSM, Active MSM in bisexuality, Sexual behaviors

## Abstract

**Background:**

Specific high-risk subgroups of men who have sex with men (MSM) play crucial roles in sexually transmitted infection (STI) transmission. This study investigated temporal trends in sexual behaviors and associated factors among bisexual MSM, active MSM, and active MSM in bisexuality.

**Methods:**

From 2010 to 2023, MSM were recruited in Zhejiang, China, through multiple strategies, including community popular opinion leader (CPOL) outreach, venue-based sampling (VBS), HIV voluntary counseling and testing (VCT) clinics, and online platforms. MSM who reported anal sex in the past six months and completed a standardized survey were classified into three high-risk subgroups. We used chi-square trend tests and weighted multivariable logistic regression analyses to assess temporal trends and associated factors. Additionally, SHapley Additive exPlanations (SHAPs) were applied to evaluate the relative importance of predictors.

**Results:**

Among the 4197 MSM, 2709 were classified as high-risk, with the proportions of the three high-risk subgroups showing gradual declines but remaining above 20%. Significantly, the rate of consistent condom use has decreased since COVID-19. Weighted logistic regression revealed that bisexual MSM were less likely to be recruited from networks (OR 0.70, 95% CI: 0.21–0.89), younger (OR 0.97, 95% CI: 0.96–0.98), and married (OR 12.68, 95% CI: 10.38–15.33). Active MSM were more likely to be recruited from public bathhouses/sauna rooms/foot spas/massage spas (OR 1.58, 95% CI: 1.32–1.88), had lower education (OR 0.70, 95% CI: 0.61–0.82), and reported lower consistent condom use (OR 0.73, 95% CI: 0.63–0.86). Engagement in commercial anal sex was associated with all three high-risk subgroups (bisexual MSM: OR 2.86, 95% CI: 2.10–3.86; active MSM: OR 1.84, 95% CI: 1.39–2.43; active MSM in bisexuality: OR 2.18, 95% CI: 1.40–3.39).

**Conclusions:**

Despite gradual declines, the proportions of high-risk MSM remain substantial, and the decline in consistent condom use since COVID-19 underscores ongoing prevention challenges. Married MSM, younger bisexual MSM, active MSM with lower education, those recruited from public venues, and individuals engaging in commercial sex represent key targets for future targeted interventions to reduce STI transmission.

**Supplementary Information:**

The online version contains supplementary material available at 10.1186/s12889-025-25517-8.

## Background

 Men who have sex with men (MSM) face a disproportionately high burden of sexually transmitted infections (STIs), including HIV, syphilis, gonorrhea, and HPV [1–6]. Globally, MSM account for approximately 23% of new HIV infections and represent the most rapidly growing HIV-infected population in China [7], increasing from 12.0% in 2010 to 23.3% in 2018 [8]. Moreover, in recent monkeypox outbreaks, 99% of male cases were MSM [9, 10], highlighting this population’s ongoing susceptibility.

Within the MSM population, specific subgroups exhibit distinct behavioral patterns that confer elevated transmission risks [11]. Bisexual MSM act as a “bridging” population between male and female sexual networks, driving infections from the MSM network into the general population [12–15]. Recent systematic reviews indicate that bisexual MSM constitute a substantial proportion of MSM in China, with increasing HIV prevalence and unique sexual behaviors that position them as a key high-risk group [16]. Active MSM, defined as those engaging in frequent anal intercourse, disproportionately increase sexual exposure within their networks, thereby heightening the risk of HIV and other STIs [14, 17, 18]. This elevated risk persists even in the context of effective biomedical interventions like antiretroviral therapy (ART) and pre-exposure prophylaxis (PrEP) [19]. Active MSM in bisexuality combine both bridging potential and high sexual activity, amplifying the breadth and intensity of transmission pathways and complicating traditional prevention strategies.

To accurately capture transmission risk, data-driven definitions were employed to classify these subgroups, focusing on recent sexual behaviors rather than self-reported sexual identity. This approach minimizes misclassification and better reflects short-term transmission risk [20]. Specifically, bisexual MSM were defined as those reporting both anal and vaginal sex in the past six months [12], and active MSM as those reporting anal sex at least once in the past week.

Previous studies have demonstrated the disproportionate burden of STIs among MSM and the heterogeneity of risk behaviors across subgroups [21–24]. However, most research has been cross-sectional or focused on specific populations, providing limited evidence of long-term temporal trends. Additionally, social and epidemiological events, including the COVID-19 pandemic, may have reshaped sexual behaviors, but reliable evidence and temporal trends on these patterns remain limited.

To address these gaps, we conducted a repeated cross-sectional study spanning 14 years to examine temporal changes in sexual behaviors among three high-risk MSM subgroups. This study aims to provide robust, actionable evidence for targeted prevention strategies and support the construction of more accurate MSM specific STI transmission models by considering the dynamics of both pre-pandemic and post-pandemic eras.

## Methods

### Study design

We conducted a repeated cross-sectional study from 2010 to 2023 among MSM in Taizhou, Zhejiang Province, China. Taizhou has long been a key site for the national HIV sentinel surveillance program targeting MSM, providing an established infrastructure for recruitment and monitoring. Although the study was conducted at a single center, the combination of multiple recruitment strategies captured diverse MSM subgroups across age, occupation, and sexual behavior profiles. This longitudinal design and broad coverage enhance the sample representativeness and allow reliable observation of temporal trends. The study aimed to characterize behavioral patterns and associated factors of three categories of high-risk MSM: bisexual MSM, active MSM, and active MSM in bisexuality.

### Recruitment strategies

Participants were recruited annually using complementary strategies to ensure broad coverage and representativeness:

- Community popular opinion leader (CPOL) recruitment: Twenty CPOLs were identified with support from local community-based organizations and Centers for Disease Control and Prevention (CDC) staff. Eligible CPOLs were socially active, well-connected, and trusted within the MSM community. They received standardized training on study recruitment procedures [25]. CPOLs mobilized community members through personal networks and organized outreach at social venues such as bars, nightclubs, and sauna rooms, distributing flyers and contact cards, referring eligible participants to the CDC study teams. The latter then obtained formal consent, performed serological testing, and conducted survey interviews.
- Venue-based sampling (VBS) recruitment: A venue-mapping exercise was conducted to identify potential recruitment sites, classified into public bathhouses/sauna rooms/foot spas/massage spas, bars/nightclubs/tea houses/clubhouses, HIV voluntary counseling and testing (VCT) clinics, and online platforms. Venues were proportionally sampled based on the estimated MSM population size. Recruitment followed three steps: mapping all potential sites, selecting monitoring venues, and approaching participants at fixed time periods [26]. Trained CDC staff provided study information, screened for eligibility, and conducted the same procedures on-site or at designated facilities. For online recruitment, advertisements were posted on MSM-focused apps and groups; interested individuals contacted the study team directly, and duplicate participation was prevented via ID verification and short screening questions.

From 2017, VCT clinic-based recruitment became a significant source. During the COVID-19 pandemic (2020 onwards), online recruitment increased due to closures and social distancing measures.

### Inclusion and exclusion criteria

Participants were eligible for inclusion if they met the following criteria: (1) Self-identified as MSM; (2) Reported engaging in anal sex in the past six months; (3) Consented to blood testing for HIV, HCV, and syphilis; (4) Signed written informed consent. Exclusion criteria were: (1) Repeat participation in the same year; (2) Incomplete questionnaire responses.

The six-month criterion for anal sex was selected to ensure the study population’s relevance to recent transmission dynamics, effectively reducing both recall and information bias, while also maintaining comparability with established MSM epidemiological studies.

### Sample size calculation

A formal sample size calculation was conducted before participant recruitment to ensure adequate statistical power for the study. Based on the proportion of MSM reporting vaginal sex with women in the past six months (*p* = 36.4%) [27], a significance level of α = 0.05, and an allowable margin of error d = 0.1 [28], the required sample size (N) was calculated using the standard formula for estimating a population proportion:

$$\mathit\:N\mathit\;\mathit=\mathit\;\mathit\;\frac{\text{Z}_{\alpha\:/2}^2\times\:\text{p}\times\:(1-\text{p})}{\text{d}^2}$$

Accounting for an anticipated 10% refusal rate, the minimum required sample size was 98 participants. Annual sample sizes ranged from 135 to 408, substantially exceeding this minimum, providing sufficient power to reliably estimate temporal trends in sexual behaviors and HIV/STI prevalence.

### Data collection and questionnaire

A structured questionnaire, developed based on the National HIV Sentinel Surveillance Implementation Plan and WHO recommendations [29], was used for data collection. Variables collected included:

- Demographic characteristics: age, marital status, education, duration of local residence, and recruitment source.
- Sexual and risk behaviors: frequency of anal sex (past week), vaginal sex (past six months), commercial anal sex (past six months), condom use (past six months), drug use (past year), and sexually transmitted diseases (STDs) diagnosed (past year).

Different recall periods were applied for behavioral variables consistent with national surveillance standards. Frequent behaviors (anal sex) were assessed over short windows to capture recent activity accurately. In contrast, less frequent behaviors (condom use, commercial sex, heterosexual activity, and STD diagnosis) used more extended recall periods to capture meaningful exposure.

### Laboratory testing

Participants provided 5 mL of venous blood, which was tested at the Taizhou CDC laboratory for HIV, HCV, and syphilis according to national protocols. HIV antibody screening was performed using the Alere HIV-1/2 kit (colloidal selenium method), with positive results confirmed by IMT HIV-1/2 Blot. Syphilis screening was conducted using TRUST and Treponema pallidum antibody ELISA kits, with positive results confirmed by TPPA. HCV testing was performed using ELISA kits, and a positive diagnosis required concordant results from two independent tests.

### Definitions of high-risk MSM

The three high risk MSM subgroups were operationally defined as follows: Bisexual MSM refers to men who have had both anal sex and vaginal sex in the past six months [30]; active MSM refers to men who have had anal sex at least once in the last week; and active MSM in bisexuality refers to bisexual men who had anal sex at least once in the last week.

### Statistical analysis

The study period was divided into three phases based on theoretical considerations and empirical evidence from joinpoint regression [31]: 2010–2016 (pre-VCT recruitment dominance), 2017–2019 (major VCT clinic integration), and 2020–2023 (COVID-19 pandemic impact) [32]. Joinpoint regression was also applied to sexual behavior indicators to identify significant trend change points, further validating the period categorization, as detailed in the Supplementary material. Within each period, descriptive statistics summarized demographic characteristics and trends in sexual behaviors of the three types of high-risk MSM. The chi-square test for trend was used to evaluate the statistical significance of observed changes over time.

To account for potential bias from varying recruitment sources [33, 34], an equal weighting scheme (1:1:1:1) was applied across the four main sources. This approach balances the contributions from each source, mitigates disproportionate influence from sources with higher recruitment in specific years, and ensures that observed trends reflect behavioral changes rather than shifts in sample composition. Weighted multivariable logistic regression was then performed to identify factors associated with the three types of high-risk MSM, both across the total sample and within each study period. SHapley Additive exPlanations (SHAP) values were calculated for the weighted logistic regression on the total sample to quantify the contribution of each independent variable to model predictions, and results were visualized using Python to enhance interpretability.

Sensitivity analyses were conducted by excluding each period in turn and repeating the weighted regression to evaluate the robustness of the results. All data were entered using EpiData 3.1 with double entry, and statistical analyses were performed in SAS 9.4 and Python 3.8.18.

## Results

**Fig. 1:**
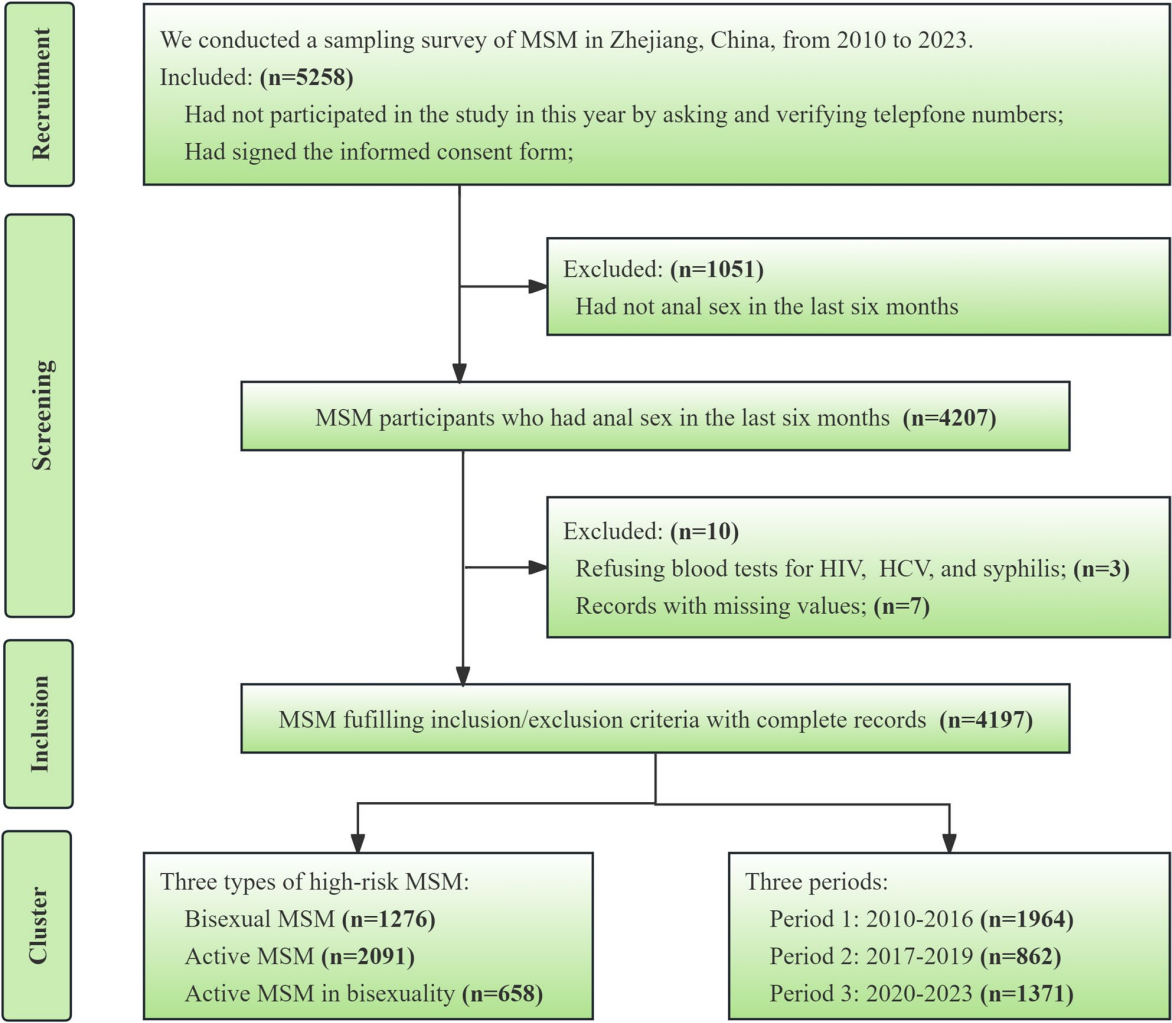
Flow chart depicting MSM recruitment, the data inclusion criteria, and cluster situation

Between 2010 and 2023, we successfully recruited 5258 MSM who provided informed consent in Zhejiang, China, and had not previously participated. The final valid sample size was 4197 MSM (Fig. 1). The filtration process involved the exclusion of 1051 participants who did not report anal sex in the past six months, three MSM who refused blood testing, and seven records with missing vital information.

### Participant demographics and recruitment sources

A total of 4197 MSM were included, with a mean age of 34.43 years (SD = 12.76). Among them, 1566 (37.3%) were married, and 1158 (27.6%) had a college education or higher. Recruitment methods included network-based recruitment (1597, 38.1%), bars/nightclubs/tea houses/clubhouses (888, 21.2%), public bathhouses/saunas/foot spas/massage spas (820, 19.5%), and VCT clinics (816, 19.4%) (Supplementary Table 1).

**Fig. 2:**
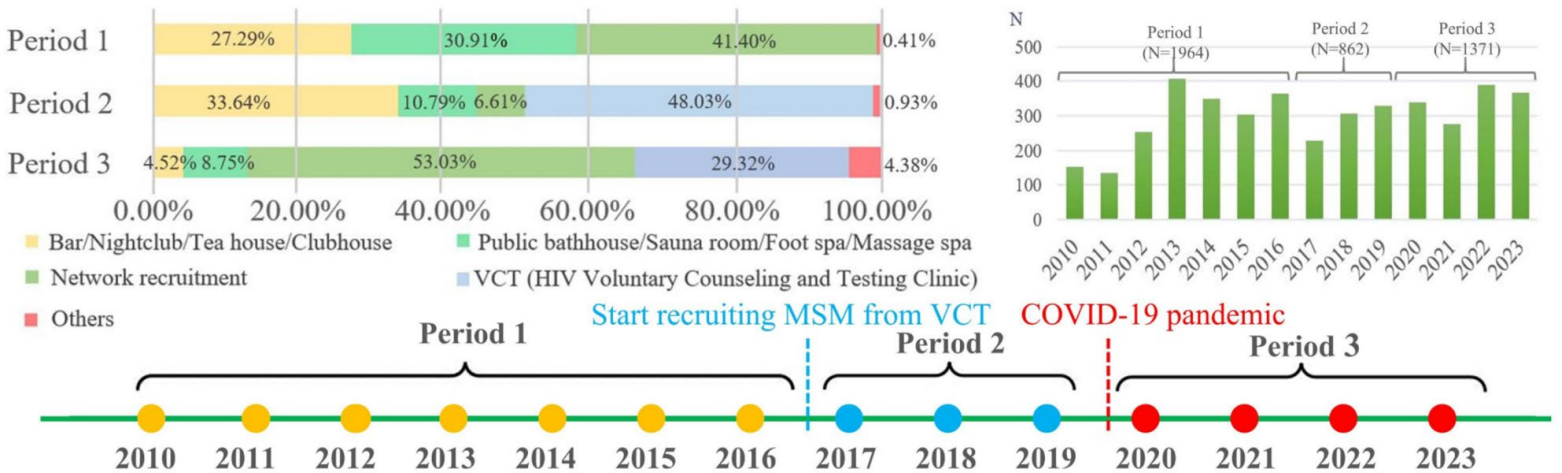
Description of the three-period divisions and the number of subjects. Due to changes in the sample sources over time, the overall data can be divided into three periods: Period 1 (2010–2016), consisting of MSM recruited from sources other than VCT clinics; Period 2 (2017–2019), consisting of MSM recruited from VCT clinics before the COVID-19 pandemic; and Period 3 (2020–2023), consisting of MSM recruited from VCT clinics after Zhejiang initiated the first-level response for the prevention and control of the COVID-19 pandemic

Across the three study periods, 1964, 862, and 1371 MSM were enrolled in Periods 1 (2010–2016), 2 (2017–2019), and 3 (2020–2023), respectively. Recruitment sources varied by period (Fig. 2): Network-based methods dominated Periods 1 and 3, while VCT clinic-based recruitment became the predominant source in Period 2, with no VCT recruitment occurring in Period 1. Detailed demographic and sexual behavior characteristics for all periods are presented in Supplementary Tables 1–3.

### Demographic characteristics of high-risk MSM

There were 1276 bisexual MSM, 2091 active MSM, and 658 active MSM in bisexuality among the participants, predominantly recruited through network recruitment and from public bathhouse/sauna rooms/foot spas/massage spas. The mean age of the high-risk MSM was 35.93 years (Table 1).

**Table 1:** Demographic characteristics among three groups of high-risk MSM

Variable	Bisexual MSM ^a^	Active MSM ^b^	Active MSM in bisexuality ^c^	High-risk MSM ^d^
*N*	1276	2091	658	2709
Sample source				
Bars/Nightclubs/Tea houses/Clubhouses	22.34% (285)	20.09% (420)	21.73% (143)	20.75% (562)
Public bathhouses/Sauna rooms/Foot spas/Massage spas	27.04% (345)	23.00% (481)	30.40% (200)	23.11% (626)
Network recruitment	31.19% (398)	35.92% (751)	29.94% (197)	35.14% (952)
VCT	18.10% (231)	19.37% (405)	16.11% (106)	19.56% (530)
Others	1.33% (17)	1.63% (34)	1,82% (12)	1.44% (39)
Age, years				
Mean ± SD	38.32 ± 11.10	35.24 ± 13.00	38.39 ± 11.23	35.93 ± 12.56
Current marital status				
Married	72.65% (927)	38.21% (799)	70.82% (466)	46.51% (1260)
Unmarried/Divorced/Widowed	27.35% (349)	61.79% (1292)	29.18% (192)	53.49% (1449)
Duration of residence				
≤ 2 years	16.77% (214)	19.32% (404)	19.60% (129)	18.05% (489)
> 2 years	83.34% (1062)	80.68% (1687)	80.40% (529)	81.95% (2220)
Education level				
Junior high school or lower	83.78% (1069)	77.00% (1610)	86.32% (568)	77.93% (2111)
College or higher	16.22% (207)	23.00% (481)	13.68% (90)	22.07% (598)

^a^ Bisexual MSM refers to men who have had both anal sex and vaginal sex in the past six months

^b^ Active MSM are those who have had anal sex at least once in the last week

^c^ Active MSM in bisexuality are those who had anal sex at least once in the last week among bisexual MSM

^d^ The number of high-risk MSM was obtained by adding bisexual MSM to active MSM and then subtracting active MSM in bisexuality

Among the samples of bisexual MSM and active MSM in bisexuality, greater percentages of married individuals were found, accounting for 72.65% (927/1276) and 70.82% (466/658), respectively, while only 38.21% (799/2091) of active MSM were married. Additionally, 23.00% (481/2091) of the active MSM had a college education or higher, while 16.22% (207/1276) and 13.68% (90/658) of the active MSM and active in bisexuality had a college education or higher, respectively (Table 1).

### Trends in behaviors and HIV/syphilis among high-risk MSM

**Fig. 3:**
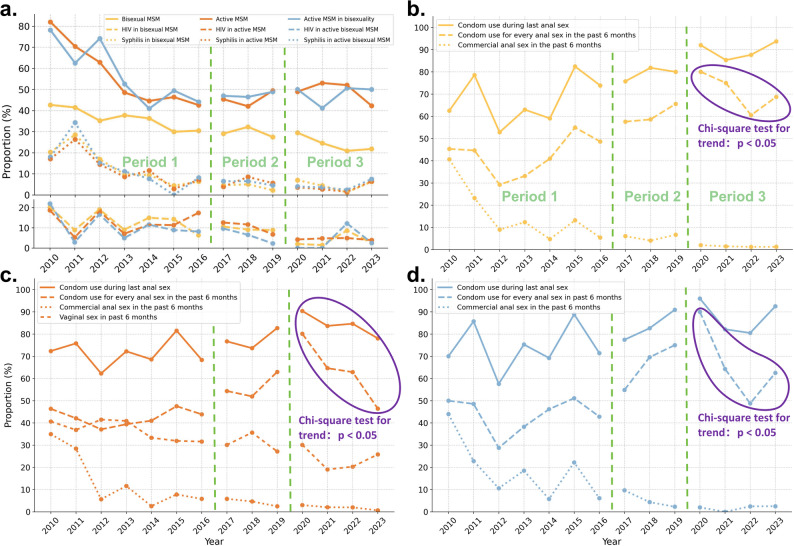
Trends in proportions, HIV/syphilis positivity, and sexual risk among high-risk MSM, 2010–2023. Panel (**a**) shows trends in the proportion and HIV/syphilis seropositivity of three high-risk MSM groups in the three periods, and panels (**b**-**d**) show trends in the proportion of different sexual risk behaviors among each group of high-risk MSM in the three periods. (Bisexual MSM refers to men who had both anal sex and vaginal sex in the past six months; active MSM were those who had anal sex at least once in the last week; active MSM in bisexuality were those who had anal sex at least once in the last week among bisexual MSM.)

We observed temporal trends in the proportions, sexual behaviors, and HIV/syphilis seropositivity among the three high-risk MSM groups across the study periods. The proportions of bisexual MSM, active MSM, and active MSM in bisexuality generally showed a decreasing trend over time but remained above 20% across the 14-year study period. The prevalence of HIV and syphilis consistently decreased over time across all three high-risk MSM groups (Fig. 3), reflecting a declining trend in STIs among these populations. Detailed annual proportions are provided in Supplementary Table 3.

Across all three periods, the proportion of the three high-risk MSM groups engaging in commercial anal sex in the past six months showed a consistent decline. Condom use during every anal sex encounter in the past six months increased in Periods 1 and 2 but decreased in Period 3. The temporal trends of condom use during the last anal sex encounter were generally consistent with those observed for every anal sex encounter over the past six months. Among active MSM, the proportion reporting vaginal sex in the past six months declined steadily throughout all three periods (Fig. 3).

### Factors related to high-risk MSM

**Fig. 4:**
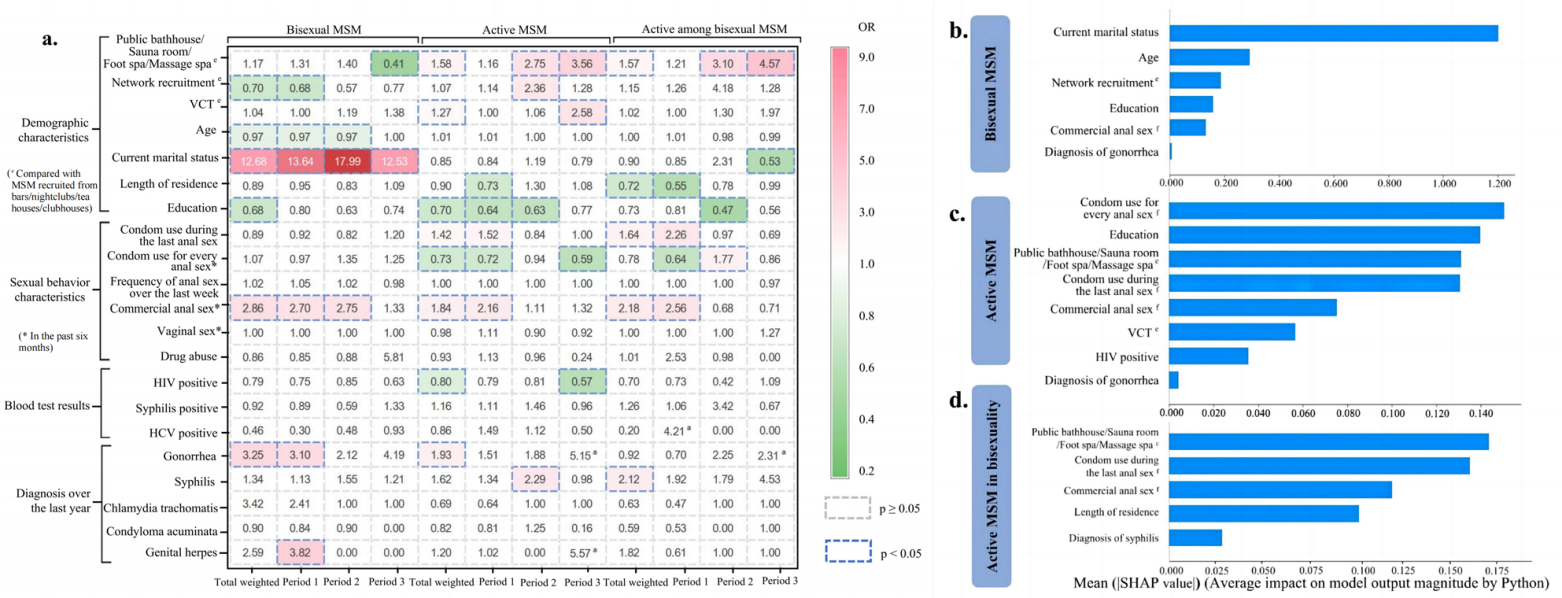
Feature importance ranking and related factors of high-risk MSM across different periods. Three high-risk MSM subgroups were analyzed: bisexual MSM (both anal and vaginal sex in the past six months), active MSM (anal sex at least once in the past week), and active MSM in bisexuality (bisexual MSM with anal sex in the past week). Data were divided into three periods to reflect missing VCT clinic data before 2016 and the COVID-19 pandemic: Period 1 (2010–2016), Period 2 (2017–2019), and Period 3 (2020–2023). Samples were weighted 1:1:1:1 by recruitment source to ensure representativeness. Weighted multivariable logistic regression was applied to all groups and to each period separately. In panel** (a)**, colors represent OR values from weighted multivariable logistic regressions. Panels** (b-c)** show SHAP plots depicting each variable’s relative contribution to model prediction; larger absolute SHAP values indicate more potent effects.

Weighted multivariable logistic regression identified several factors associated with different high-risk MSM subgroups (Fig. 4). Detailed statistical results are presented in the Supplementary Tables 4–6.

- For bisexual MSM, key factors included network recruitment, younger age, lower education level, current marital status, engaging in commercial anal sex, and recent gonorrhea diagnosis.
- For active MSM, the associated factors were recruitment from public bathhouses/sauna rooms/foot spas/massage spas and VCT, lower education, engaging in commercial anal sex, condom use during anal sex, HIV test positivity, and recent gonorrhea diagnosis.
- For active MSM in bisexuality, the related factors included local residence for more than two years, recruitment from public bathhouses/sauna rooms/foot spas/massage spas, condom use in the last anal sex, engaging in commercial anal sex, and recent syphilis diagnosis.

To further assess factor importance, we calculated SHAP values. For bisexual MSM, marital status was the most influential factor (OR 12.68, 95% CI: 10.38–15.33), followed by age (OR 0.97, 95% CI: 0.96–0.98) and network recruitment (OR 0.70, 95% CI: 0.21–0.89). Among active MSM, the highest contributions were observed for consistent condom use during anal sex (OR 1.42, 95% CI: 1.19–1.72), education level (OR 0.70, 95% CI: 0.61–0.82), and recruitment from public bathhouses/sauna rooms/foot spas/massage spas (OR 1.58, 95% CI: 1.32–1.88), which displayed relatively similar SHAP values. For active MSM in bisexuality, the two most important factors were recruitment from public bathhouses/sauna rooms/foot spas/massage spas (OR 1.57, 95% CI: 1.13–2.16) and condom use during the last anal sex encounter (public bathhouses/sauna rooms/foot spas/massage spas; OR 1.63, 95% CI: 1.25–2.12).

Subgroup analyses stratified by study period revealed dynamic changes in associated factors. Among bisexual MSM, marital status consistently demonstrated a strong positive association (all ORs > 10) across all three periods. Commercial anal sex was significant in Periods 1 and 2 but not in Period 3. For active MSM, consistent condom use was not significant in Period 2 but showed a protective effect in Periods 1 and 3. Education was associated in Periods 1 and 2 but lost significance in Period 3. Furthermore, recruitment from public bathhouses/sauna rooms/foot spas/massage spas was associated with Periods 2 and 3, but not in Period 1. Finally, among active MSM in bisexuality, recruitment from public bathhouses/sauna rooms/foot spas/massage spas was associated in Periods 2 and 3, with progressively increasing effect sizes over time.

### Sensitivity analysis

The sensitivity analysis was conducted by excluding each period in turn, assigning weights of 1:1:1:1 according to the sample source, and performing weighted multivariable logistic regression to observe any changes in the results. The sensitivity analyses did not show any significant changes, and detailed results are presented in the Supplementary Fig. 1.

## Discussion

Our 14-year repeated cross-sectional study provides the first comprehensive assessment of long-term trends in high-risk MSM proportions and sexual behaviors across three distinct periods, encompassing 4197 participants. Despite an overall decreasing trend in the proportions of bisexual MSM, active MSM, and active MSM in bisexuality, these high-risk groups consistently remained above 20% throughout the study. Although the relative proportion is declining, the increasing total number of MSM means that the absolute risks posed by this population remain substantial and warrant continued public health attention [35].

A key finding regarding sexual behavior was the decreased consistent condom use (during every anal sex encounter in the past six months) among all high-risk MSM groups during the COVID-19 pandemic (Period 3). This observation is consistent with other studies, including a modeling study that estimated a 25% reduction in condom use consistency in China during the pandemic [36]. This decline is most plausibly attributed to pandemic-related restrictions: strict lockdown measures and limitations on social gatherings reduced opportunities for new sexual encounters and access to physical venues where MSM typically meet partners [37]. This reduction in high-risk mixing opportunities likely altered the dynamics of partner trust. It thus influenced consistent condom use patterns, a finding supported by a nationwide survey in China documenting substantial declines in multiple sexual partners and mobility for sexual activity among MSM during local epidemic periods [38].

SHAP-based weighted multivariable logistic regression identified key determinants of high-risk MSM, highlighting the critical role of individual characteristics and recruitment venues. For bisexual MSM, being married and younger were the most influential factors [39], suggesting that marital status and age could serve as key screening signals; targeted interventions could include confidential counseling, routine VCT services [40], and accessible online self-testing kits. For active MSM, inconsistent condom use and lower educational attainment are important, emphasizing the need for tailored health education. This education should utilize plain language, visual aids, and short videos via social media, alongside distribution of condoms and on-site rapid testing. Recruitment venue further differentiated these risk profiles [33, 34]: public bathhouses/sauna rooms/foot spas/massage spas were strongly associated with active MSM, whereas bars/nightclubs/tea houses/clubhouses were linked to bisexual MSM. These findings indicate that preventive measures should be implemented in a subgroup-specific manner, guided by behavioral characteristics, to enhance the effectiveness of interventions. Importantly, by combining long-term repeated cross-sectional data with SHAP values, this study provides a strong, evidence-driven framework for understanding temporal trends in MSM behaviors and informs targeted public health strategies for these high-risk populations.

Our subgroup analyses reveal that the relative importance of behavioral determinants among MSM is dynamic across the study periods. For bisexual MSM, the association of commercial sex weakened in Period 3. This is likely a consequence of the reduced prevalence of this behavior since COVID-19 [38], which results in wider confidence intervals and a loss of statistical significance. Concurrently, the greater reliance on recruitment from specific sources during the pandemic may have masked commercial sex’s independent contribution. Furthermore, for active MSM, consistent condom use demonstrated a negative association in Periods 1 and 3, but a positive one in Period 2, while the association with educational attainment also weakened in Period 3. Collectively, these findings highlight that bs, which in turn influence the risks associated with these subgroups. This underscores the critical need for interventions that are subgroup-specific, responsive to temporal changes, and sustained beyond short-term prevention efforts.

To our knowledge, this is the first repeated cross-sectional study to comprehensively describe behavioral patterns and trends among high-risk MSM over 14 years. By objectively defining three high-risk subgroups, with bisexual MSM identified based on recent anal and vaginal sex and active MSM based on anal sex frequency, we provide a more accurate reflection of STI risk than relying on self-identification. Subgroup analyses across study periods further offer insights into temporal changes in behaviors, supporting targeted and behavior-informed prevention strategies.

Nonetheless, several limitations warrant consideration. Data were collected from a single location, which inherently limits their generalizability, and self-reported behaviors may be subject to social desirability bias. In addition, our equal weighting approach assumes comparable representativeness across recruitment sources and cannot fully reconstruct the actual population distribution. Despite these constraints, our weighting approach provides a transparent method to standardize trends, ensuring that observed changes more likely reflect behavioral shifts rather than sample composition. These limitations highlight the need for cautious interpretation and guide directions for future, more representative research.

## Conclusions

Over the 14 years, while the proportions of high-risk MSM subgroups have gradually declined, they remain substantial. Crucially, consistent condom use has declined since the COVID-19 pandemic, indicating persistent STI risk. Determinants vary by subgroup and period, including marital status, age, education, condom use, and recruitment venues. These findings underscore the need for sustained, evidence-based, subgroup-specific interventions, including venue-targeted outreach, tailored sexual health education, vigorous promotion of consistent condom use, and accessible HIV/STI testing with online self-testing options.

## Supplementary Information

Supplementary Material 1.

## Data Availability

The fully anonymized dataset is available from Haijiang Lin on reasonable request.

## Abbreviations

MSM: Men who have sex with men
STIs: Sexually transmitted infections
STDs: Sexually transmitted diseases
ART: Antiretroviral therapy
PrEP: Pre-exposure prophylaxis
CPOL: Community popular opinion leader
VBS: Venue-based sampling
VCT: Voluntary counseling and testing
CDC: Centers for Disease Control and Prevention
SHAP: SHapley Additive exPlanations
