# Supplementary material for: Trends of sexual behaviors and related factors in high-risk MSM from 2010 to 2023: a repeated cross-sectional study in Zhejiang, China
Source: BMC Public Health. 2025 Dec 18;25:4264. doi: 10.1186/s12889-025-25517-8 (PMC12713285; doi:10.1186/s12889-025-25517-8)
Supplement: Supplementary file 1 — Supplementary Material 1. [file 12889_2025_25517_MOESM1_ESM.docx]

**Appendix to:**

**Trends of sexual behaviors and related factors in high-risk MSM: a repeated cross-sectional study from 2010 to 2023 in Zhejiang, China**

authors

**Contents**

The results of the joinpoint analysis of sexual behaviors for the period division (Supplementary Figure 1-4) [2](#bookmark1)

Supplementary Table 1. Demographic characteristics among MSM participants in different periods……………….[5](#bookmark1)

Supplementary Table 2. Demographic characteristics among MSM from 2010 to 2023 …………………………………. [6](#bookmark2)

Supplementary Table 3. Sexual behavior characteristics among MSM from 2010 to 2023 …………………………..….[7](#bookmark3)

Supplementary Table 4. Multifactor logistic regression results of bisexual MSM among participants

in different periods (α= 0.05) …………………………………………………………………………………………………………………………[8](#bookmark4)

Supplementary Table 5. Multifactor logistic regression results of active MSM among participants

in different periods (α= 0.05) ………………………………………………………………………………………………………………………….9

Supplementary Table 6. Multifactor logistic regression results of active MSM in bisexuality in

different groups (α= 0.05) ……………………………………………………………………………………………………………………………...1[0](#bookmark6)

Supplementary Figure 5. Sensitivity analysis of factors associated with three types of high-risk MSM

among participants in different groups ………………………………………………………………………………………………………….. [1](#bookmark7)1

Questionnaire (English)……………………………………………………………………………………………………………………………………..12

**The results of the joinpoint analysis of sexual behaviors for the period division**

We conducted joinpoint regression analysis to assess whether statistically significant change-points in sexual behavior indicators supported our original period definitions. We used Joinpoint regression software (version 5.4.0, available through the Surveillance Research Program of the United States National Cancer Institute), as described by Kim et al. This method evaluates both the magnitude and direction of temporal trends, detects statistically significant joinpoints where trend changes, and estimates separate slopes between these points. The analysis starts with zero joinpoints and sequentially tests whether adding up to two joinpoints significantly improves model fit.
Model selection was based on the distribution and scale of the dependent variables. When annual proportions were relatively high and approximated a normal distribution, a linear model (y = Xb) was applied. These included:
(1) Proportion of MSM who reported condom use during their last anal sex.
(2) Proportion of MSM who reported consistent condom use during anal sex in the past 6 months.
(3) Proportion of MSM who reported heterosexual intercourse in the past 6 months.
When annual proportions were small and better suited to an exponential or Poisson distribution, a log-linear model (ln(y) = Xb) was applied, as in the case of the proportion of MSM who reported commercial anal sex during the past 6 months.
Among all indicators analyzed, only the proportion of MSM who reported consistent condom use during anal sex in the past 6 months showed two statistically significant joinpoints, in 2017 and 2020. For this indicator, the estimated trends corresponded closely to our pre-defined study periods: 2010–2016, 2017–2019, and 2020–2023.

**
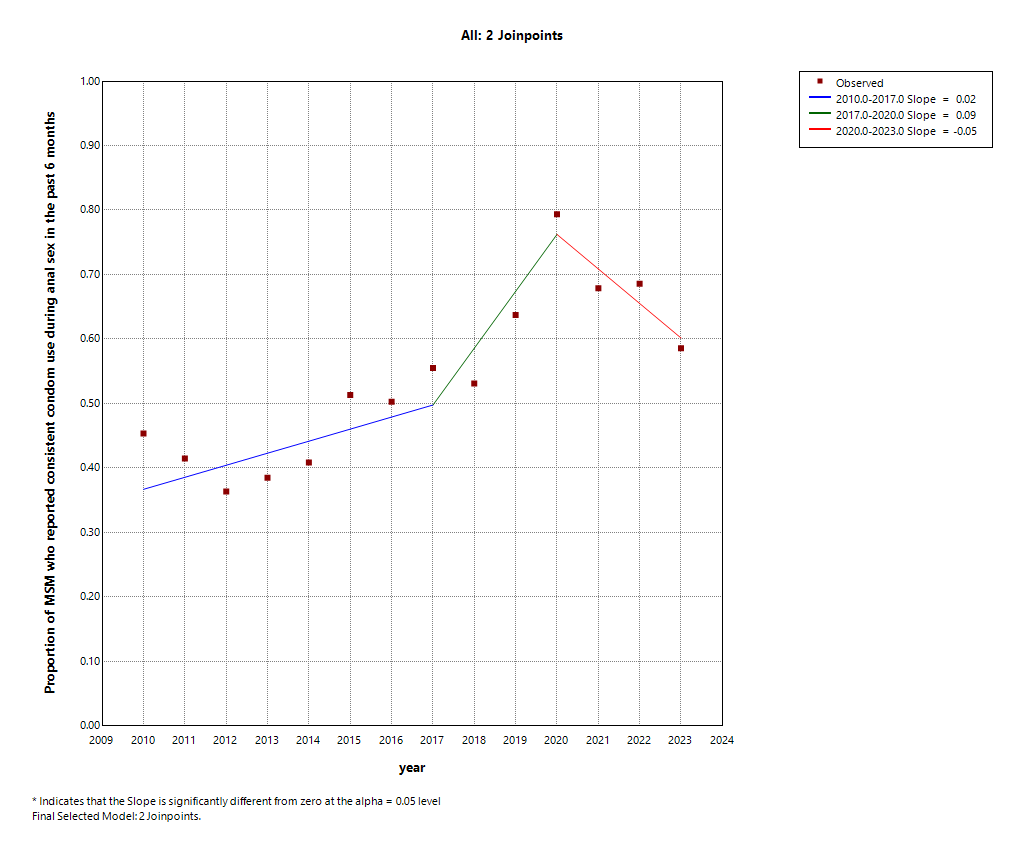
**

**Supplementary Figure 1. The results of the joinpoint analysis of the proportion of MSM who reported consistent condom use during anal sex in the past 6 months.**

**
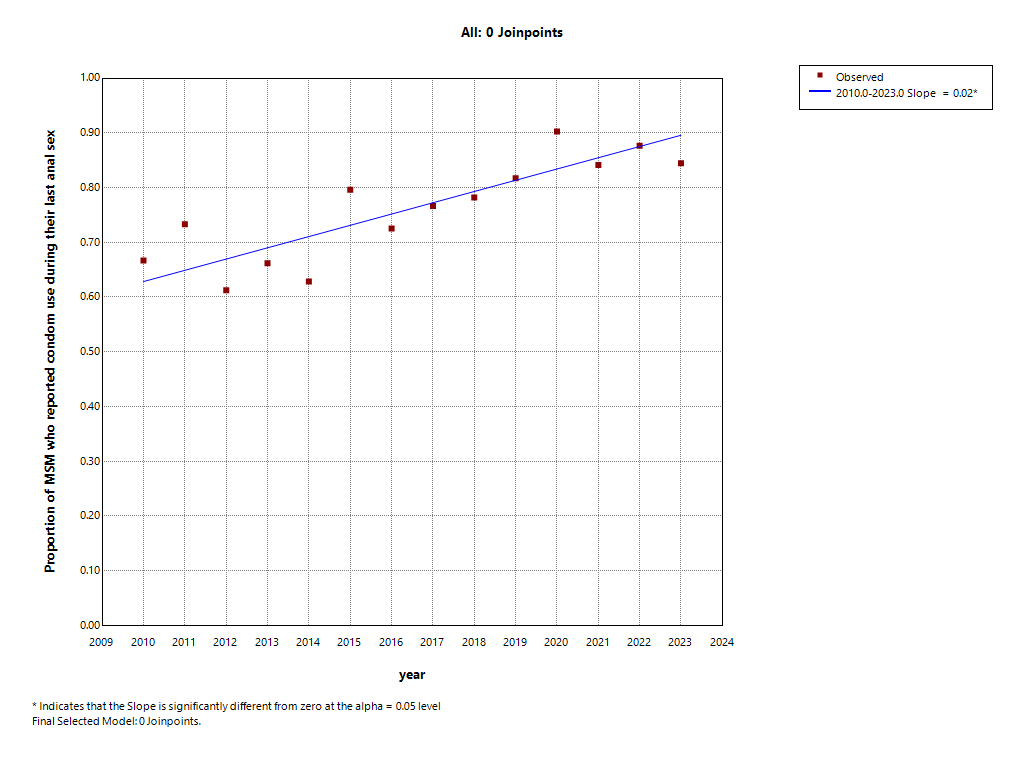
**

**Supplementary Figure 2. The results of the joinpoint analysis of the proportion of MSM who reported condom use during their last anal sex.**

**
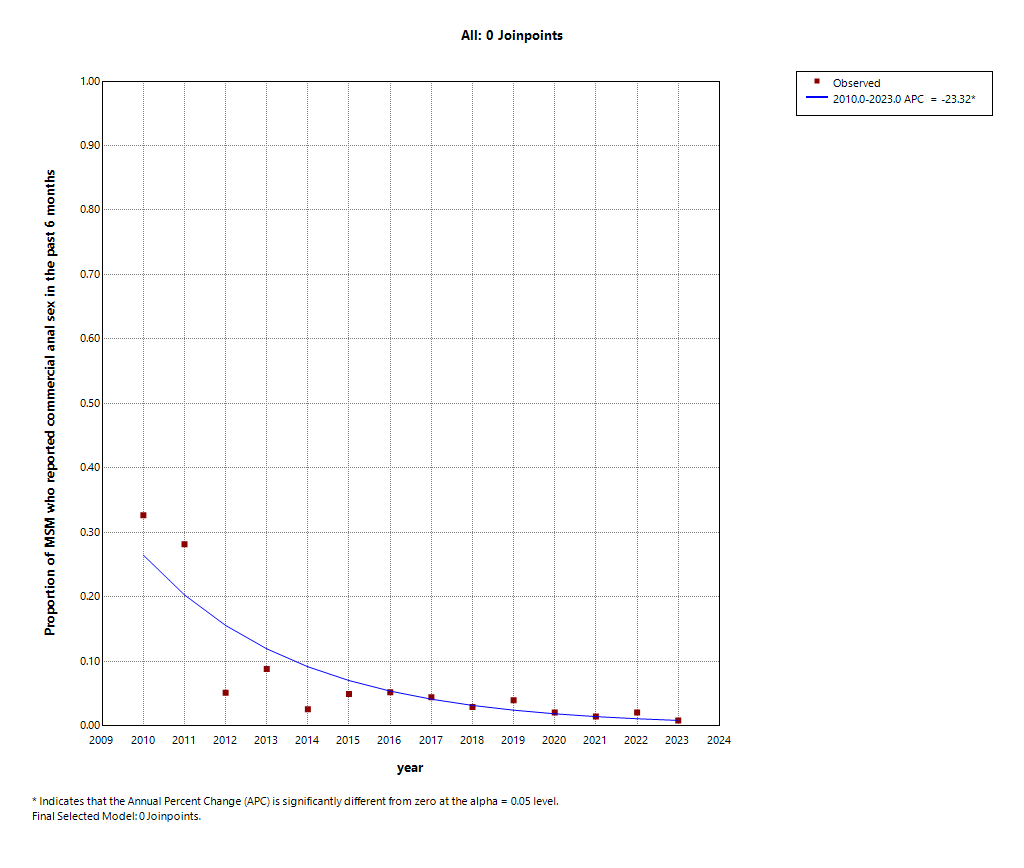
**

**Supplementary Figure 3. The results of the joinpoint analysis of the proportion of MSM who reported commercial anal sex during the past 6 months**

**
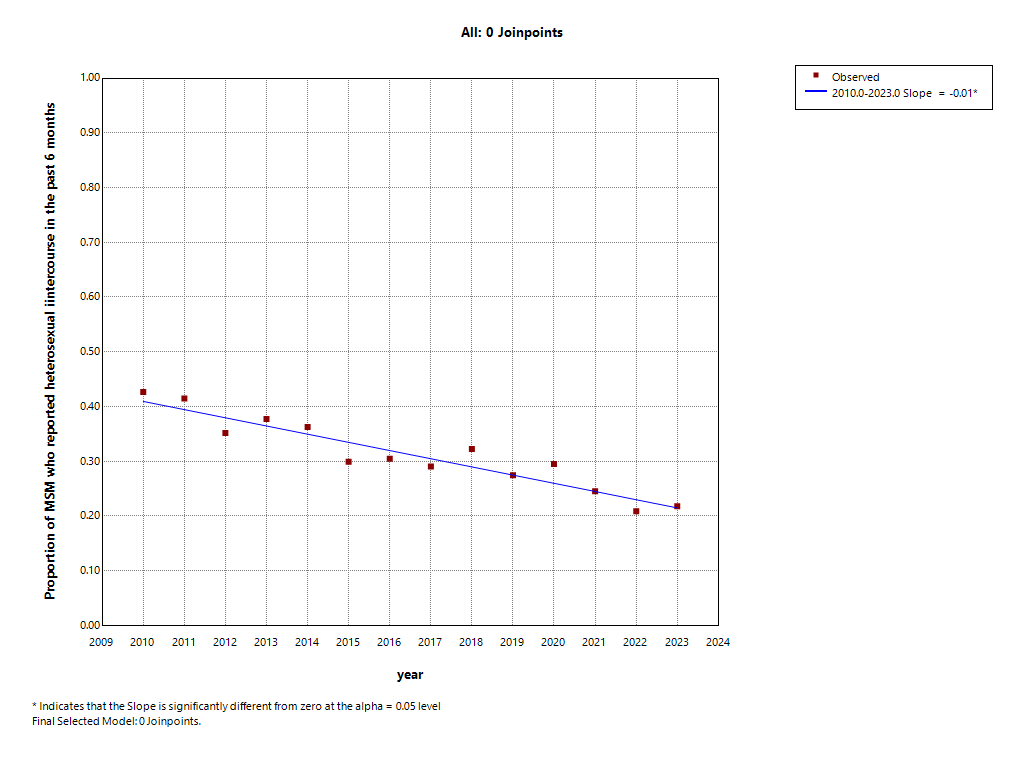
**

**Supplementary Figure 4. The results of the joinpoint analysis of the proportion of MSM who reported heterosexual intercourse in the past 6 months.**

**Supplementary Table 1.** **Demographic characteristics among MSM participants in different periods**

| **Variable** | **Total** | **V^a^** | χ²/F | **p value** | **Period 1**  **（2010-2016）** | **Period 2**  **（2017-2019）** | **Period 3**  **（2020-2023）** |
| --- | --- | --- | --- | --- | --- | --- | --- |
| **N** | 4197 | — | — | — | 1964 | 862 | 1371 |
| **Sample source** |  | 0.30 | 778.26^b^ | <0.001 |  |  |  |
| Bar/Nightclub/Tea house/Clubhouse | 21.16% (888) |  |  |  | 27.29% (536) | 33.64% (290) | 4.52% (62) |
| Public bathhouse/Sauna room/Foot spa/Massage spa | 19.54% (820) |  |  |  | 30.91% (607) | 10.79% (93) | 8.75% (120) |
| Network recruitment | 38.05% (1597) |  |  |  | 41.40% (813) | 6.61% (57) | 53.03% (727) |
| VCT (HIV Voluntary Counseling and Testing Clinic) | 19.44% (816) |  |  |  | 0 | 48.03% (414) | 29.32% (402) |
| Others | 1.81% (76) |  |  |  | 0.41% (8) | 0.93% (8) | 4.38% (60) |
| **Age, years** |  | — | 2.69^c^ | 0.07 |  |  |  |
| Mean±SD | 34.43 ± 12.76 |  |  |  | 34.59 ± 13.01 | 33.54 ± 12.11 | 34.75 ± 12.78 |
| **Current marital status** |  | 0.06 | 13.37^b^ | 0.001 |  |  |  |
| Married | 37.31% (1566) |  |  |  | 39.86% (784) | 37.12% (320) | 33.70% (462) |
| Unmarried /Divorced /Widowed | 62.69% (2631) |  |  |  | 60.14% (1183) | 62.88% (542) | 66.30% (909) |
| **Length of residence** |  | 0.07 | 21.26^b^ | <0.001 |  |  |  |
| ≤ 2 years | 25.11% (1054) |  |  |  | 21.40% (421) | 15.89% (137) | 15.83% (217) |
| > 2 years | 74.89% (3143) |  |  |  | 78.60% (1546) | 84.11% (725) | 84.17% (1154) |
| **Education** |  | 0.10 | 45.10^b^ | <0.001 |  |  |  |
| Junior high school or lower | 27.59% (1158) |  |  |  | 77.28% (1520) | 69.81% (602) | 67.10% (920) |
| College or higher | 72.41% (3039) |  |  |  | 22.72% (447) | 30.16% (260) | 32.90% (451) |

^a^ V is a statistical indicator to measure the difference between variables in the Chi-square test. Larger values indicate a greater degree of variation.

(V =$\sqrt{\chi^{2}}/\sqrt{N\cdot\min\left( k-1,r-1 \right)}$, V is Cramér's V, *N* is the total number of observations, *k* is the number of groups, and *r* is the number of categories.)

^b^ Chi-square test is performed for categorical variables in demographic characteristics by subgroup.

^c^ Analysis of Variance is employed for the continuous variables in demographic characteristics by subgroup, and F is the statistic.

**Supplementary Table 2.** **Demographic characteristics among MSM from 2010 to 2023**

| **Variable** | **Period 1** | | | | | | |  | **Period 2** | | |  | **Period 3** | | | |
| --- | --- | --- | --- | --- | --- | --- | --- | --- | --- | --- | --- | --- | --- | --- | --- | --- |
|  | **2010** | **2011** | **2012** | **2013** | **2014** | **2015** | **2016** |  | **2017** | **2018** | **2019** |  | **2020** | **2021** | **2022** | **2023** |
| **N** | 150 | 135 | 253 | 408 | 350 | 304 | 364 |  | 227 | 307 | 328 |  | 339 | 277 | 388 | 367 |
| **Sample source** |  |  |  |  |  |  |  |  |  |  |  |  |  |  |  |  |
| Bar/Nightclub/Tea house/Clubhouse | 53.95% | 15.56% | 16.21% | 28.92% | 19.71% | 31.25% | 30.68% |  | 37.89% | 40.72% | 24.09% |  | 12.98% | 2.89% | 2.58% | 0 |
| Public bathhouse/Sauna room/  Foot spa/Massage spa | 6.58% | 37.78% | 40.71% | 39.95% | 46.57% | 20.07% | 15.62% |  | 5.29% | 13.68% | 11.89% |  | 13.27% | 14.08% | 7.33% | 1.63% |
| Network recruitment | 39.47% | 46.67% | 43.08% | 30.39% | 33.71% | 48.68% | 52.33% |  | 1.76% | 1.30% | 14.94% |  | 28.32% | 22.38% | 71.13% | 79.84% |
| VCT ^a^ | 0 | 0 | 0 | 0 | 0 | 0 | 0 |  | 55.07% | 43.00% | 47.87% |  | 37.17% | 60.65% | 16.49% | 11.99% |
| Others | 0 | 0 | 0 | 0.74% | 0 | 0 | 1.37% |  | 0 | 1.30% | 1.21% |  | 8.26% | 0 | 2.06% | 6.54% |
| **Age, years** |  |  |  |  |  |  |  |  |  |  |  |  |  |  |  |  |
| Mean | 28.41 | 35.22 | 36.33 | 36.66 | 37.13 | 33.87 | 31.52 |  | 31.49 | 34.41 | 34.15 |  | 36.28 | 35.44 | 34.21 | 33.39 |
| SD | 8.24 | 11.69 | 13.57 | 13.33 | 14.57 | 12.61 | 11.6 |  | 10.26 | 12.83 | 12.46 |  | 13.77 | 13.77 | 13.44 | 9.88 |
| **Current marital status** |  |  |  |  |  |  |  |  |  |  |  |  |  |  |  |  |
| Married | 23.03% | 46.67% | 47.04% | 45.59% | 42.86% | 33.22% | 35.62% |  | 38.33% | 41.04% | 32.62% |  | 39.53% | 32.49% | 31.44% | 31.61% |
| Unmarried /Divorced /Widowed | 76.97% | 53.33% | 52.96% | 54.41% | 57.14% | 66.78% | 64.38% |  | 61.67% | 58.96% | 67.38% |  | 60.47% | 67.51% | 68.56% | 68.39% |
| **Length of residence** |  |  |  |  |  |  |  |  |  |  |  |  |  |  |  |  |
| ≤ 2 years | 41.45% | 20.00% | 20.55% | 20.10% | 16.29% | 23.18% | 18.63% |  | 14.54% | 15.64% | 17.07% |  | 13.86% | 11.91% | 14.69% | 21.80% |
| > 2 years | 58.55% | 80.00% | 79.45% | 79.90% | 83.71% | 76.82% | 81.37% |  | 85.46% | 84.36% | 82.93% |  | 86.14% | 88.09% | 85.31% | 78.20% |
| **Education** |  |  |  |  |  |  |  |  |  |  |  |  |  |  |  |  |
| Junior high school or lower | 76.97% | 83.70% | 84.19% | 81.13% | 78.00% | 74.34% | 67.67% |  | 68.72% | 71.66% | 68.90% |  | 71.68% | 66.06% | 68.81% | 61.85% |
| College or higher | 23.03% | 16.30% | 15.81% | 18.87% | 22.00% | 25.66% | 32.33% |  | 31.28% | 28.34% | 31.10% |  | 28.32% | 33.94% | 31.19% | 38.15% |

^a^ HIV Voluntary Counseling and Testing Clinic

**Supplementary Table 3. Sexual behavior characteristics among MSM from 2010 to 2023**

| **Variable** | **Period 1** | | | | | | |  | **Period 2** | | |  | **Period 3** | | | |
| --- | --- | --- | --- | --- | --- | --- | --- | --- | --- | --- | --- | --- | --- | --- | --- | --- |
|  | **2010** | **2011** | **2012** | **2013** | **2014** | **2015** | **2016** |  | **2017** | **2018** | **2019** |  | **2020** | **2021** | **2022** | **2023** |
| **N** | 150 | 135 | 253 | 408 | 350 | 304 | 364 |  | 227 | 307 | 328 |  | 339 | 277 | 388 | 367 |
| **Frequency of anal sex in the past week** | |  |  |  |  |  |  |  |  |  |  |  |  |  |  |  |
| Mean | 2.63 | 1.31 | 0.96 | 0.75 | 0.66 | 0.76 | 0.78 |  | 0.87 | 0.67 | 0.73 |  | 0.66 | 0.81 | 0.71 | 0.58 |
| SD | 2.66 | 1.45 | 1.14 | 1.17 | 1.13 | 1.32 | 2.29 |  | 0.19 | 1.14 | 0.94 |  | 0.85 | 1.17 | 0.89 | 0.88 |
| **Condom use for every anal sex in the past six months** | | |  |  |  |  |  |  |  |  |  |  |  |  |  |  |
| Yes | 45.33% | 41.48% | 36.36% | 38.48% | 40.86% | 51.32% | 50.27% |  | 55.51% | 53.09% | 63.72% |  | 79.35% | 67.87% | 68.56% | 58.58% |
| No | 55.26% | 58.52% | 63.64% | 61.52% | 59.14% | 48.68% | 49.59% |  | 44.49% | 46.91% | 36.28% |  | 20.65% | 32.13% | 31.44% | 41.42% |
| **Commercial anal sex in the past six months** | |  |  |  |  |  |  |  |  |  |  |  |  |  |  |  |
| Yes | 32.89% | 28.15% | 5.14% | 8.82% | 2.57% | 4.93% | 5.22% |  | 4.41% | 2.93% | 3.96% |  | 2.06% | 1.44% | 2.06% | 0.82% |
| No | 67.55% | 71.85% | 8.82% | 91.18% | 4.93% | 95.07% | 4.41% |  | 95.59% | 97.07% | 96.04% |  | 97.94% | 98.56% | 97.94% | 99.18% |
| **Vaginal sex in the past six months** | |  |  |  |  |  |  |  |  |  |  |  |  |  |  |  |
| Yes | 42.67% | 41.48% | 35.18% | 37.75% | 36.29% | 29.93% | 30.49% |  | 29.07% | 32.25% | 27.44% |  | 29.50% | 24.55% | 20.88% | 21.80% |
| No | 57.89% | 58.52% | 64.82% | 62.25% | 63.71% | 70.07% | 69.59% |  | 79.93% | 67.75% | 72.56% |  | 70.50% | 75.45% | 79.12% | 78.20% |
| **Positive blood test results** |  |  |  |  |  |  |  |  |  |  |  |  |  |  |  |  |
| HIV | 20.00% | 10.37% | 17.00% | 10.05% | 14.00% | 12.83% | 15.38% |  | 9.69% | 11.40% | 10.98% |  | 4.42% | 6.14% | 4.64% | 3.27% |
| Syphilis | 18.67% | 24.44% | 13.44% | 9.07% | 10.86% | 5.26% | 6.87% |  | 3.69% | 6.51% | 4.27% |  | 4.42% | 3.25% | 1.03% | 4.36% |
| HCV | 0.67% | 0 | 0.40% | 0.49% | 0.29% | 0.33% | 0 |  | 0 | 0.33% | 0.91% |  | 0.88% | 0.36% | 0.52% | 0.27% |

**Supplementary Table 4. Multifactor logistic regression results of bisexual MSM among participants in different periods (α= 0.05)**

| **Variables** | **Total weighted ^c^** | | | **Period 1** | | | | **Period 2** | | | | **Period 3** | | | |
| --- | --- | --- | --- | --- | --- | --- | --- | --- | --- | --- | --- | --- | --- | --- | --- |
|  | **b (95%CI)** | **χ²** | **p value** | **b (95%CI)** | **χ²** | **p value** | | **b (95%CI)** | **χ²** | | **p value** | **b (95%CI)** | | **χ²** | **p value** |
| **Sample source ^a^** |  |  |  |  |  | |  |  |  | |  |  | |  |  |
| Public bathhouse/Sauna room/Foot spa/Massage spa | 0.16 (-0.07- 0.39) | 1.96 | 0.161 | 0.27 (-0.04- 0.59) | 2.89 | | 0.089 | 0.34 (-0.27- 0.94) | 1.19 | | 0.203 | -0.90 (-1.71- -0.08) | | 4.68 | 0.031* |
| Network recruitment | -0.35 (-0.57- -0.12) | 9.38 | 0.002* | -0.38(-0.66- -0.11） | 7.41 | | 0.007* | -0.57 (-1.34 -0.21) | 2.06 | | 0.275 | -0.26 (-0.98- 0.45) | | 0.52 | 0.470 |
| VCT ^d^ | 0.04 (-0.18- 0.26） | 0.13 | 0.717 | — | — | | — | 0.17 (-0.23- 0.58) | 0.70 | | 0.151 | 0.32 (-0.41- 1.05) | | 0.73 | 0.394 |
| **Age** | -0.03 (-0.04- -0.02) | 56.40 | < 0.001* | -0.03 (-0.04- -0.02) | 30.33 | | <.0.001* | -0.03 (-0.05- -0.02) | 12.34 | | < 0.001* | | 0.00 (-0.02- 0.01) | 0.16 | 0.686 |
| **Current marital status** | 2.54 (2.34- 2.73) | 645.72 | <.0.001* | 2.61 (2.33- 2.88) | 339.18 | | < 0.001* | 2.89 (2.42- 3.36) | 144.40 | | < 0.001* | | 2.53 (2.22- 2.84) | 254.90 | < 0.001* |
| **Length of residence** | -0.12 (-0.32- 0.08) | 1.30 | 0.254 | -0.05 (-0.32- 0.23) | 0.11 | | 0.743 | -0.19 (-0.68- 0.29) | 0.62 | | 0.431 | 0.09 (-0.36- 0.54） | | 0.15 | 0.696 |
| **Education** | -0.38 (-0.58- -0.17) | 13.13 | <.0.001* | -0.22 (-0.51- 0.07) | 2.15 | | 0.142 | -0.47 (-0.93- -0.01) | 4.00 | | 0.046 | -0.31 (-0.68- 0.07） | | 2.60 | 0.107 |
| **Condom use during the last anal sex** | -0.12 (-0.34- 0.10) | 1.18 | 0.278 | -0.08 (-0.36- 0.20) | 0.31 | | 0.579 | -0.20 (-0.73- 0.34) | 0.52 | | 0.469 | 0.19 (-0.38- 0.75) | | 0.42 | 0.516 |
| **Condom use for every anal sex ^b^** | 0.07 (-0.12- 0.26) | 0.59 | 0.444 | -0.03(-0.23- 0.30) | 0.06 | | 0.805 | 0.30 (-0.14- 0.75) | 1.77 | | 0.184 | 0.23 (-0.17- 0.62) | | 1.26 | 0.262 |
| **Commercial anal sex ^b^** | 1.05 (0.74- 1.35) | 44.55 | < 0.001* | 0.99 (0.63- 1.35) | 28.83 | | < 0.001* | 1.01 (0.17- 1.84) | 5.58 | | 0.018* | 0.28 (-0.87- 1.43) | | 0.23 | 0.630 |
| **Frequency of anal sex in the past week** | 0.02 (-0.04- 0.08) | 0.51 | 0.477 | 0.05 (-0.03- 0.12) | 1.59 | | 0.207 | 0.02 (-0.14- 0.18) | 0.08 | | 0.783 | -0.02 (-0.19- 0.14) | | 0.08 | 0.774 |
| **Drug abuse** | -0.15 (-0.31- 0.01) | 3.19 | 0.074 | -0.16 (-0.94- 0.63) | 0.15 | | 0.702 | -0.13 (-0.34- 0.08) | 1.58 | | 0.208 | 1.76 (-0.39-3.91) | | 2.57 | 0.109 |
| **Diagnosis of STD in the past year** |  |  |  |  |  | |  |  |  | |  |  | |  |  |
| Gonorrhea | 1.18 (0.39- 1.97) | 8.55 | 0.004* | 1.13 (0.15- 2.11) | 5.11 | | 0.024* | 0.75 (-1.16- 2.67) | 0.59 | | 0.442 | 1.43 (-2.07- 4.94) | | 0.64 | 0.423 |
| Syphilis | 0.29 (-0.18- 0.75) | 1.45 | 0.228 | 0.12 (-0.50- 0.75) | 0.15 | | 0.699 | 0.44 (-0.57- 1.46) | 0.73 | | 0.393 | 0.19 (-1.20- 1.59) | | 0.07 | 0.785 |
| Genital chlamydia trachomatis infection | 1.23 (-0.53- 2.99) | 1.87 | 0.172 | 0.88 (-0.76- 2.52) | 1.11 | | 0.292 | — | | — | — | — | | — | — |
| Condyloma acuminatum | -0.10 (-0.87- 0.67) | 0.07 | 0.796 | -0.18 (-1.00- 0.65) | 0.17 | | 0.678 | -0.10 (-2.02- 1.83) | 0.01 | | 0.919 | -12.85(-1134.60- 1108.90) | | 0.00 | 0.982 |
| Genital herpes | 0.95 (-0.41- 2.31) | 1.86 | 0.172 | 1.34 (0.02- 2.65) | 3.99 | | 0.046* | -10.00(-992.80-972.80) | | 0.00 | 0.984 | -14.59(-2915.30-2886.10) | | 0 .00 | 0.992 |
| **Positive blood test results** |  |  |  |  |  | |  |  |  | |  |  | |  |  |
| HIV | -0.24 (-0.49- -0.01) | 3.47 | 0.063 | -0.29 (-0.62- 0.03) | 3.12 | | 0.077 | -0.16 (-0.76- 0.44) | 0.27 | | 0.601 | -0.47 (-1.24- 0.30) | | 1.42 | 0.232 |
| Syphilis | -0.08 (-0.36- 0.20) | 0.29 | 0.587 | -0.12 (-0.48- 0.23) | 0.46 | | 0.497 | -0.53 (-1.39- 0.33) | 1.47 | | 0.225 | 0.29 (-0.47- 1.04) | | 0.55 | 0.458 |
| HCV | -0.78 (-1.93- 0.38) | 1.74 | 0.187 | -1.19 (-3.51- 1.14) | 1.00 | | 0.318 | -0.74 (-3.91-2.42) | 0.21 | | 0.645 | -0.07 (-1.80-1.67) | | 0.01 | 0.939 |

^a^ Take the source from bar/cabaret/tearoom/club as reference ^b^ in the past six months ^c^ Weighted according to sample sources of aggregate data

^d^ HIV Voluntary Counseling and Testing Clinic ^*^ p < 0.05

**Supplementary Table 5. Multifactor logistic regression results of active MSM among participants in different periods (α= 0.05)**

| **Variables** | **Total weighted ^c^** | | | | **Period 1** | | | **Period 2** | | | | | **Period 3** | | | |
| --- | --- | --- | --- | --- | --- | --- | --- | --- | --- | --- | --- | --- | --- | --- | --- | --- |
|  | **b (95%CI)** | | **χ²** | **p value** | **b (95%CI)** | **χ²** | **p value** | **b (95%CI)** | | **χ²** | **p value** | | **b (95%CI)** | | **χ²** | **p value** |
| **Sample source ^a^** |  | |  |  |  |  |  |  | |  | |  |  | |  |  |
| Public bathhouse/Sauna room/Foot | 0.46 (0.28- 0.63) | | 24.98 | < 0.001* | 0.15 (-0.12- 0.42) | 1.15 | 0.283 | 1.01 (0.52- 1.51) | | 16.00 | < 0.001* | | 1.27 (0.62-1.91) | | 14.73 | < 0.001* |
| spa/Massage spa |  | |  |  |  |  |  |  | |  |  | |  | |  |  |
| Network recruitment | 0.07 (-0.10-0.25) | | 0.67 | 0.414 | 0.13 (-0.10- 0.37) | 1.31 | 0.252 | 0.86 (0.27- 1.45) | | 8.19 | | 0.004 * | | 0.25 (-0.30- 0.80) | 0.79 | 0.373 |
| VCT ^d^ | 0.24 (0.06- 0.41) | | 6.74 | 0.009* | — | — | — | 0.06 (0.261- 0.38) | | 0.14 | | 0.704 | 0.95 (0.39- 1.51) | | 10.88 | 0.001* |
| **Age** | 0.01 (0.00- 0.01) | | 2.69 | 0.101 | 0.01 (0.00-0.02) | 2.19 | 0.139 | 0.00 (-0.01- 0.01) | | 0.05 | | 0.824 | 0.00 (-0.01- 0.01) | | 0.01 | 0.944 |
| **Current marital status** | -0.16 (-0.33- 0.02) | | 3.13 | 0.077 | -0.18(-0.43-0.07) | 1.97 | 0.160 | 0.17 (-0.24- 0.57) | | 0.63 | | 0.424 | -0.24 (-0.56- 0.08) | | 2.17 | 0.140 |
| **Time of residence** | -0.10 (-0.26- 0.07) | | 1.28 | 0.258 | -0.32(-0.55- -0.10) | 8.06 | 0.005* | | 0.26 (-0.13- 0.65) | 1.68 | | 0.195 | 0.08 (-0.24- 0.40) | | 0.24 | 0.623 |
| **Education** | -0.35 (-0.50- -0.20) | | 21.48 | < 0.001* | -0.45(-0.67 -0.24) | 16.78 | < 0.001* | | -0.46 (-0.77- -0.14) | 8.16 | | 0.004* | -0.26 (-0.52- 0.00) | | 3.88 | 0.049 |
| **Condom usage during the last anal sex** | 0.35 (0.17- 0.54) | | 14.77 | < 0.001* | 0.42 (0.19-0.66) | 12.49 | < 0.001* | | -0.17 (-0.59- 0.26) | 0.57 | | 0.449 | 0.00 (-0.41- 0.441 | | 0.00 | 0.994 |
| **Regular condom usage during anal sex ^b^** | | -0.31 (-0.46- -0.15) | 15.19 | < 0.001* | -0.33(-0.55- -0.10) | 8.37 | 0.004* | | -0.06 (-0.41- 0.30) | 0.11 | | 0.742 | -0.53 (-0.77- -0.28) | | 17.78 | < 0.001* |
| **Commercial anal intercourse ^b^** | 0.61 (0.33- 0.89) | | 18.50 | < 0.001* | 0.77 (0.43-1.11) | 20.05 | < 0.001* | | 0.10 (-0.64- 0.84) | 0.07 | | 0.795 | 0.28 (-0.60- 1.16) | | 0.39 | 0.534 |
| **Vaginal sex ^b^** | -0.02 (-0.17- 0.06) | | 0.05 | 0.816 | 0.10 (-0.12-0.32) | 0.78 | 0.379 | -0.10 (-0.46- 0.25) | | 0.33 | | 0.563 | -0.08 (-0.38- 0.23) | | 0.25 | 0.619 |
| **Drug usage** | -0.07(-0.21- 0.44) | | 1.27 | 0.261 | 0.12(-0.54- 0.77) | 0.13 | 0.722 | -0.04 (-0.13- 0.20) | | 0.20 | | 0.655 | -1.44 (-3.74- 0.86) | | 1.51 | 0.219 |
| **Diagnosis of STD in the past year** |  | |  |  |  |  |  |  | |  | |  |  | |  |  |
| Gonorrhea | 0.66 (0.13- 1.44) | | 5.94 | 0.015* | 0.41 (-0.54- 1.35) | 0.72 | 0.396 | 0.63(-1.13-2.40) | | 0.50 | | 0.481 | 13.15 (-1006.60-1032.90) | | 0.00 | 0.980 |
| Syphilis | 0.48 (-0.07- 0.90) | | 5.27 | 0.022 | 0.29 (-0.24- 0.83) | 1.17 | 0.279 | 0.83 (0.11- 1.77) | | 2.96 | | 0.039* | -0.02 (-1.00- 0.97) | | 0.00 | 0.976 |
| Genital chlamydia trachomatis infection | -0.37 (-1.80- 1.06) | | 0.25 | 0.614 | -0.44 (-1.81- 0.93) | 0.40 | 0.529 | — | | — | | — | — | | — | — |
| Condyloma acuminatum | -0.20 (-0.80- 0.40) | | 0.44 | 0.509 | -0.21(-0.86- 0.45) | 0.38 | 0.539 | 0.22 (-1.31- 1.74) | | 0.08 | | 0.780 | -1.85 (-4.11- 0.42) | | 2.55 | 0.11 |
| Genital herpes | 0.18 (-1.05- 1.40) | | 0.08 | 0.777 | 0.02 (-1.23- 1.28) | 0.00 | 0.969 | -12.36 (-892.30-867.60) | | 0.00 | | 0.978 | 13.23 (-1430.20- 1456.70) | | 0.00 | 0.986 |
| **Positive blood test results** |  | |  |  |  |  |  |  | |  | |  |  | |  |  |
| HIV | -0.22 (-0.42- -0.02) | | 4.59 | 0.032* | -0.24(-0.51- 0.03) | 3.04 | 0.081 | -0.21 (-0.68-0.26) | | 0.79 | | 0.375 | -0.56 (-1.11- -0.02) | | 4.06 | 0.044* |
| Syphilis | 0.15 (-0.09- 0.39) | | 1.47 | 0.226 | 0.10 (-0.20- 0.41) | 0.45 | 0.501 | 0.38 (-0.28- 1.05) | | 1.28 | | 0.258 | -0.04 (-0.69- 0.62) | | 0.01 | 0.910 |
| HCV | -0.15 (-1.04- 0.73) | | 0.11 | 0.737 | 0.40 (-1.33- 2.13) | 0.21 | 0.650 | 0.11 (-1.98- 2.20) | | 0.01 | | 0.915 | -0.68 (-2.20- 0.84) | | 0.78 | 0.378 |

^a^ Take the source from bar/cabaret/tearoom/club as reference ^b^ in the past six months ^c^ Weighted according to sample sources of aggregate data

^d^ HIV Voluntary Counseling and Testing Clinic ^*^ p < 0.05

**Supplementary Table 6. Multifactor logistic regression results of active MSM in bisexuality in different groups (α= 0.05)**

|  | **Total weighted ^c^** | | | | | **Period 1** | | | | | | **Period 2** | | | | | **Period 3** | | |
| --- | --- | --- | --- | --- | --- | --- | --- | --- | --- | --- | --- | --- | --- | --- | --- | --- | --- | --- | --- |
| **Variables** | **b (95%CI)** | | **χ²** | **p value** | | **b (95%CI)** | | **χ²** | **p value** | | | | **b (95%CI)** | **χ²** | **p value** | | **b (95%CI)** | **χ²** | **p value** |
| **Sample source ^a^** |  | |  |  | |  | |  |  | | |  | |  |  | |  |  |  |
| Public bathhouse/Sauna room/  Foot spa/Massage spa | 0.45 (0.12- 0.77) | | 7.27 | 0.007* | | 0.19 (-0.26- 0.64) | | 0.69 | 0.405 | | | 1.13 (0.28- 1.98) | | 6.72 | 0.010* | | 1.52 (0.20- 2.83) | 5.13 | 0.024* |
| Network recruitment | 0.14 (-0.19- 0.47) | | 0.69 | 0.406 | | 0.23 (-0.19- 0.65) | | 1.17 | 0.279 | | | 1.43 (0.09- 2.78) | | 4.38 | 0.036 | | 0.25 (-0.90- 1.41) | 0.18 | 0.669 |
| VCT ^d^ | 0.02 (-0.32- 0.35) | | 0.01 | 0.923 | | — | | — | — | | | 0.26 (-0.37- 0.88) | | 0.66 | 0.417 | | 0.68 (-0.51- 1.87) | 1.26 | 0.262 |
| **Age** | 0.00 (-0.01- 0.01) | | 0.05 | 0.832 | | 0.01 (-0.01- 0.02) | | 0.31 | 0.580 | | | -0.02 (-0.06- 0.01) | | 1.65 | 0.200 | | -0.01 (-0.04- 0.02) | 0.73 | 0.395 |
| **Current marital status** | -0.10 (-0.40- 0.20) | | 0.44 | 0.506 | | -0.16 (-0.57- 0.26) | | 0.55 | 0.460 | | | 0.84 (0.07- 1.61) | | 4.55 | 0.033 | | -0.63 (-1.16- -0.10) | 5.40 | 0.020* |
| **Length of residence** | -0.33 (-0.64- -0.02) | | 4.36 | 0.037* | | -0.60 (-1.01- 0.19) | | 8.23 | 0.004* | | | -0.25 (-1.04- 0.54) | | 0.39 | 0.535 | | -0.37 (-0.41- 1.14) | 0.86 | 0.353 |
| **Education** | -0.32 (-0.64- 0.00) | | 3.75 | 0.053 | | -0.21 (-0.66- 0.25) | | 0.79 | 0.376 | | | -0.76 (-1.51- -0.03) | | 4.12 | 0.042* | | -0.15 (-0.78- 0.47) | 0.24 | 0.621 |
| **Condom use during the last anal sex** | 0.49 (0.22- 0.75) | | 12.94 | < 0.001* | | | 0.81 (0.41- 1.21) | 15.86 | < 0.001* | | | | -0.03 (-0.90- 0.84) | 0.00 | 0.953 | | -0.03(-0.97- 0.90) | 0.00 | 0.946 |
| **Condom use for every anal sex ^b^** | -0.25 (-0.54- 0.03) | | 3.00 | 0.083 | | | -0.44 (-0.82- 0.05) | 4.98 | 0.026* | | | | 0.57 (0.03- 1.11) | 4.27 | 0.039* | | -0.34 (-0.96-0.29) | 1.13 | 0.288 |
| **Commercial anal sex ^b^** | | 0.78 (0.34- 1.22) | 12.04 | 0.001* | | 0.94 (0.43- 1.46) | | 12.77 | < 0.001* | | | | -0.39 (-0.83- 1.61) | 0.40 | 0.529 | | 0.24 (-1.67- 2.16) | 0.06 | 0.804 |
| **Drug abuse** | 0.01 (-0.23- 0.25) | | 0.01 | 0.941 | | 0.93 (-0.48- 2.33) | | 1.66 | 0.197 | | | | -0.02 (-0.33- 0.29) | 0.02 | 0.902 | | -15.36(-2760.00-2729.20) | < 0.01 | 0.991 |
| **Diagnosis of gonorrhea in the past year** |  | |  |  | |  | |  |  | | |  | |  |  | |  |  |  |
| Gonorrhea | -0.08 (-1.22- 1.06) | | 0.02 | 0.889 | | -0.36(-1.66- 0.94) | | 0.29 | 0.589 | | | 0.81 (-2.14- 3.76) | | 0.29 | 0.591 | | 14.65(-3918.30-3947.60) | 0.00 | 0.994 |
| Syphilis | 0.75 (0.07- 1.43) | | 4.66 | 0.031* | | 0.65 (-0.21- 1.51) | | 2.19 | 0.139 | | | 0.58 (-0.90- 2.05) | | 0.59 | 0.443 | | 1.51 (-0.93- 3.96) | 1.48 | 0.225 |
| Genital chlamydia trachomatis infection | -0.46 (-2.55- 1.17) | | 0.22 | 0.642 | | -0.76 (-2.76- 1.24) | | 0.56 | 0.455 | | | — | | — | — | | — | — | — |
| Condyloma acuminatum | -0.53 (-2.38- 1.47) | | 0.93 | 0.335 | | -0.63 (-1.94- 0.69) | | 0.86 | 0.353 | | | -13.05 (-1696.10- 1670.0) | | 0.00 | 0.988 | | — | — | — |
| Genital herpes | 0.60 (-1.82-0.62) | | 0.01 | 0.921 | | -0.49 (-2.29- 1.30) | | 0.29 | 0.591 | | — | | | — | — | | — | — | — |
| **Positive blood test results** |  | |  |  | |  | |  |  | | |  | |  |  | |  |  |  |
| HIV | -0.35 (-0.74- 0.05) | | 3.00 | 0.083 | | -0.31 (-0.79- 0.16) | | 1.69 | 0.193 | | | -0.86 (-1.88- 0.16) | | 2.72 | 0.099 | | 0.09 (-1.14- 1.32) | 0.02 | 0.887 |
| Syphilis | 0.23 (-0.20- 0.65) | | 1.10 | 0.295 | | 0.06 (-0.45- 0.56) | | 0.05 | 0.827 | | | 1.23 (-0.23- 2.69) | | 2.72 | 0.099 | | -0.40 (-1.55-0.75) | 0.46 | 0.498 |
| HCV | -1.60 (-4.01- 0.82) | | 1.67 | 0.196 | 12.95(-1439.40-1465.30) | | | < 0.01 | | 0.986 | | -16.05 (-2399.70-2367.60) | | < 0.01 | | 0.990 | -16.19(-2796.10-2763.70) | < 0.01 | 0.991 |

^a^ Take the source from bar/cabaret/tearoom/club as reference ^b^ in the past six months ^c^ Weighted according to sample sources of aggregate data

^d^ HIV Voluntary Counseling and Testing Clinic ^*^ p < 0.05


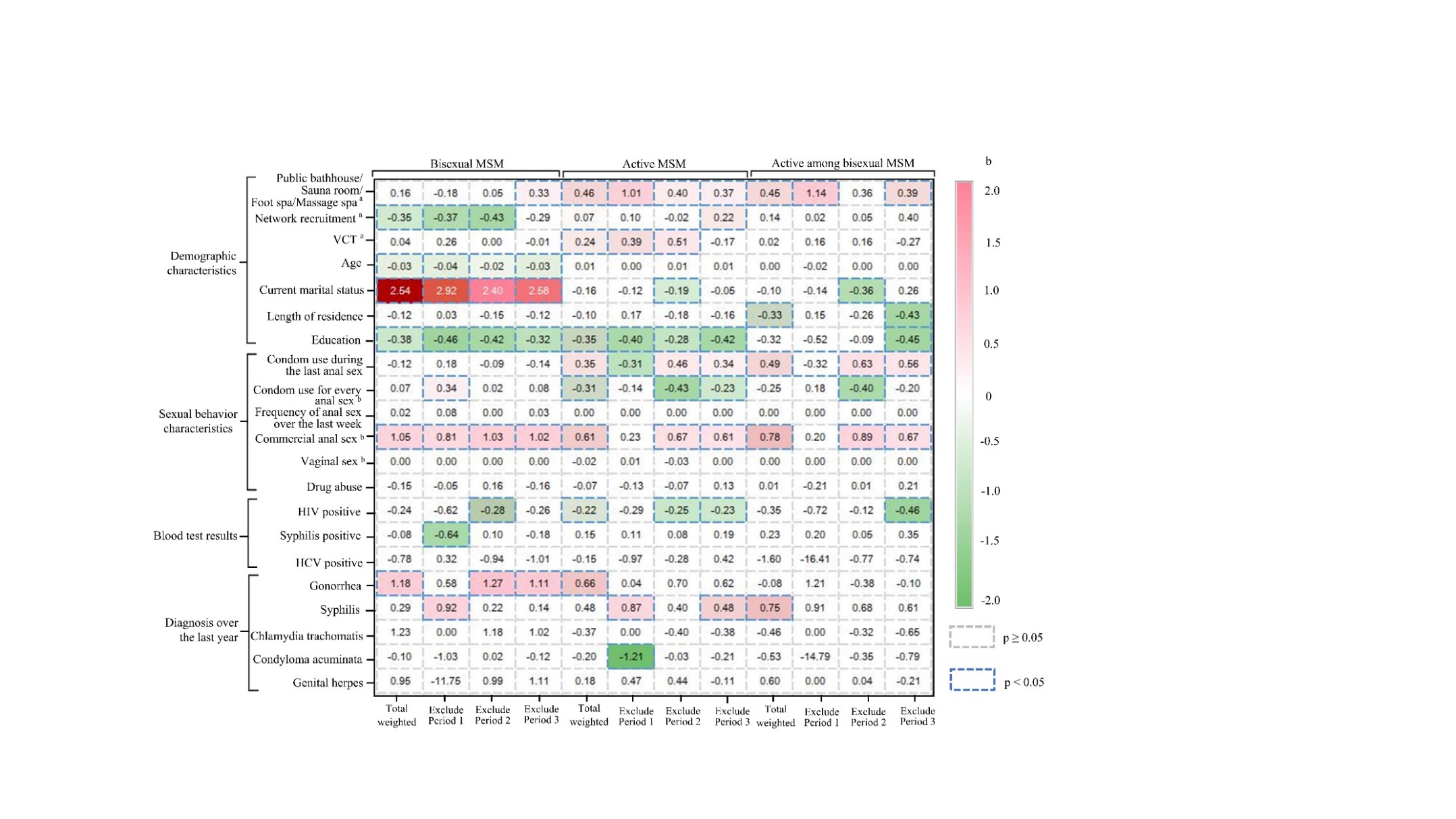
**Supplementary Figure 5. Sensitivity analysis of factors associated with three types of high-risk MSM among participants in different groups**

We investigated factors associated with three categories of high-risk MSM: bisexual MSM, active MSM, and MSM active in bisexuality. Bisexual MSM refers to men who have had both anal sex and vaginal sex in the past six months; Active MSM are those who have had anal sex at least once in the last week among men who have had anal sex in the past six months; Active MSM in bisexuality are those who had anal sex at least once in the last week among men who had had both anal sex and vaginal sex in the past six months. The figure displays the results of the sensitivity analysis for these high-risk MSM categories. Due to the absence of VCT source data before 2016 and the impact of COVID-19, we divided the sample into three periods: Period 1 (2010-2016), Period 2 (2017-2019), and Period 3 (2020-2023). To make our MSM sample more representative, we combined the data in a 1:1:1:1 ratio based on sample sources and weighted accordingly. The sensitivity analysis was conducted. Specifically, we excluded each period in turn, assigned weights of 1:1:1:1 according to the sample source (excluding other sources), and performed weighted multifactor logistic regression to observe any changes in results. Each cell in the graph represents the regression coefficient (b) of relevant variables, with red indicating a positive correlation, green indicating a negative correlation, and white indicating no correlation. The number within each cell represents the value of the regression coefficient (b). (^a^ Compared with MSM samples from bar/cabaret /tearoom/club sources; ^b^ In the past six months.)

### MSM questionnaire (English)

**Basic information**

A01 Monitoring location:

A02 No.: □□□（001—999）

A03Date : □□□□ (Year), □□ (Month), □□ (Day)

A04 Sample source: □

① Bar/Nightclub/Tea house/Clubhouse

② Public bathhouse/Sauna room/Foot spa/Massage spa

③ Network recruitment

④ VCT (HIV Voluntary Counseling and Testing Clinic)

⑤ Others

**Hi, my name is ...... and I am from ....... We are surveying to find out the state of people's knowledge and behavior on a number of health issues. Please be assured that this survey is anonymous, and we will keep your answers confidential. We want your answers to reflect your true individuality. The survey will take about 10 minutes of your time, and at the end, I can provide you with some assistance (e.g., you can ask some health questions, and I will try my best to answer them). I hope you will support our work. Thank you!**

**May I ask if you have recently participated in this survey? If the answer is "yes," then we will conclude this visit.**

**Demographic information**

B01 Birth year:

B02 Current marital status: □ ①Married ②Unmarried /Divorced /Widowed

B03 Length of residence □ ① ≤ 2 years ② > 2 years

B04 Education □ ① Junior high school or lower ② College or higher

**Sexual behaviors information**

C01 In the last six months, have you had anal sex with men?

□ ① Yes ② No (skip to D01) ③ Refuse to answer

C02 In the last week, how many times have you had anal sex with men? Times/per week

C03 In the last six months, did you use condoms during your last anal sex act with men?

□ ① Yes ② No ③ Refuse to answer

C04 In the last six months, did you use condoms every time you had anal sex with men?

□ ① Yes ② No ③ Refuse to answer

D01 In the last six months, have you had commercial sex with men?

□ ① Yes ② No (skip to E01) ③ Refuse to answer

D02 In the last six months, did you use condoms during your last commercial anal sex act with men?

□ ① Yes ② No ③ Refuse to answer

D03 In the last six months, did you use condoms every time you had commercial anal sex with men?

□ ① Yes ② No ③ Refuse to answer

E01 In the last six months, have you had sex with a woman?

□ ① Yes ② No (skip to F01) ③ Refuse to answer

E02 In the last six months, did you use condoms every time you had vaginal sex with women?

□ ① Yes ② No ③ Refuse to answer

E03 In the last six months, did you use condoms every time you had vaginal sex with women?

□ ① Yes ② No ③ Refuse to answer

**Other behaviors**

F01 Are you a drug addict?

□ ① Yes ② No ③ Refuse to answer

**Sexually transmitted diseases**

G01 In the last year, have you been diagnosed with any of the following sexually transmitted diseases (gonorrhea, syphilis, genital chlamydia trachomatis infection, condyloma acuminatum, and genital herpes)?

□ ① Yes ② No (skip to H01) ③ Refuse to answer

G02 In the last year, have you been diagnosed with gonorrhea?

□ ① Yes ② No ③ Refuse to answer

G03 In the last year, have you been diagnosed with syphilis?

□ ① Yes ② No ③ Refuse to answer

G04 In the last year, have you been diagnosed with genital chlamydia trachomatis infection?

□ ① Yes ② No ③ Refuse to answer

G05 In the last year, have you been diagnosed with condyloma acuminatum?

□ ① Yes ② No ③ Refuse to answer

G06 In the last year, have you been diagnosed with genital herpes?

□ ① Yes ② No ③ Refuse to answer

**The survey concludes here. Thank you for your cooperation. To assess your health status, we need to collect blood for HIV, syphilis, and HCV testing.**

**Blood test**

H01 Does this survey consent to blood collection for testing?

□ ① Yes ② No (skip to end survey)

H02 The first ELISA screening HIV antibody test result

□ ① Positive ② Negative (skip to H04)

H03 The second ELISA confirmatory HIV antibody test result

□ ① Positive ② Negative (skip to H04)

H04 The ELISA screening for syphilis test result

□ ① Positive ② Negative (skip to H06)

H05 The RPR/TRUST screening syphilis test result

□ ① Positive ② Negative (skip to H06)

H06 The first ELISA screening HCV antibody test result

□ ① Positive ② Negative (skip to end survey)

H07 The second ELISA confirmatory HCV antibody test result

□ ① Positive ② Negative

**Investigator's signature: Supervisor's signature:**
